# How professional capital and team heterogeneity affect the demands of online team-based medical service

**DOI:** 10.1186/s12911-019-0831-y

**Published:** 2019-06-28

**Authors:** Jiaying Li, Hong Wu, Zhaohua Deng, Naiji Lu, Richard Evans, Chenxi Xia

**Affiliations:** 1 School of Medicine and Health Management, Tongji Medical College, Huazhong University of Science and Technology, 13 Hangkong Road, Qiaokou District, Wuhan, China; 2 College of Engineering, Design and Physical Sciences, Brunel University London, London, UK

**Keywords:** Online healthcare communities, Online medical teams, Team-based service demands, Professional capital, Team heterogeneity

## Abstract

**Background:**

The provision of medical services by Medical Teams (MT) on Online Healthcare Communities (OHCs) is a novel method employed by geographically-dispersed healthcare professionals to serve one patient simultaneously, allowing patients to receive more specific, targeted and comprehensive advice. As a relatively new method of service delivery, little attention has been paid to identifying the determinants of Team-based Service Demands (TSD). Based on Upper Echelons Theory and Social Exchange Theory, this study examines the impact of both professional capital (*status capital* and *decisional capital*) and team heterogeneity (*team size* and *dispersion*) on TSD.

**Methods:**

This study uses data collected from 890 MTs, employing 3994 team members, operating on haodf.com, a Chinese OHC, to examine effects of both leader and team characteristics on TSD.

**Results:**

Our findings suggest that a MT’s characteristics have a significant impact on TSD. Firstly, the decisional capital of both leaders and teams were positively related with TSD, while only the status capital of leaders saw a positive impact. Secondly, team heterogeneity influenced TSD in two ways: (1) provided a direct negative impact and (2) positively moderated the relationship between professional capital and TSD.

**Conclusion:**

This paper comprehensively studies the impact of TSD from the perspectives of professional capital and team heterogeneity, expanding current theoretical understanding of team heterogeneity and social capital in OHCs. Further, it provides practical suggestions for platform development and team leaders managing MTs in online environments.

## Background

The continued growth in web-mediated communication and collaboration has created considerable interest in online virtual teams [1], without exception in the healthcare industry. Over the past two decades, healthcare providers have been encouraged to collaborate more frequently with individuals and teams outside of their departmental boundaries, with the aim of improving healthcare provision worldwide [2], and satisfying the varying demands of citizens. Since 2017, medical teams have emerged in China on OHCs, such as Haodf.com (https://www.haodf.com/) and Guahao.com (https://www.guahao.com/).

MTs are traditionally composed of a Founder (called a leading specialist), and other medical professional members, such as doctors and consultants, who operate in different departments, hospitals, or regions around the world. By collaboratively serving patients online, the key stakeholders of MTs, including doctors and patients, receive numerous benefits. Firstly, team-based service provision provides an opportunity for members to become better known by others, extending the professional network of MT members. Secondly, by collaborating with doctors from other departments, hospitals or regions, strong social bonds can be established, enabling the exchange of personal experiences and lessons learnt during co-discussion [3]. Third, MTs are able to improve patients’ trust and decrease information asymmetry, as advice received from more than one doctor can reduce patients’ uncerntainty of e.g. diagnosis or advice provided [4, 5]. In summary, team-based service provision enables more informed decisions to be made by doctors, through consultation with other professionals worldwide, and a reduction in patient uncertainty during diagnosis and recovery.

Before the emergence of MTs, medical consultation was delivered as a one-to-one service with physicians, resulting in extended wait times to receive e.g. medical results, which were often bias, due to only receiving advice or diagnosis from one doctor. Presently, patients still lack effective channels for accessing medical services from multiple doctors simultaneously, with existing service providers on OHCs experiencing numerous challenges, including: (1) doctors being engaged with many patients at the same time; it is often difficult for doctors to balance their workload between online and offline service delivery, and (2) some doctors on OHCs receive less patients due to being considered too young, having a perceived insufficient background or for other reasons. Given the imbalance and polarization in online medical services, it is necessary that hospitals allocate doctors and their time reasonably. On the other hand, citizen demands for high-quality healthcare and increasing trust issues between doctors and patients put pressure on traditional medical service delivery [6, 7]. Due to the powerful synthesized capital of team members, online MTs have the potential to provide services with higher quality for patients [8], while mitigating pressure, as well as sharing the work of doctors on OHCs. As a means for improving healthcare services online, MTs have drawn intense attention of the industry [9].

Existing studies have examined the determinants of one-to-one service demands on OHCs, as well as the service demands of medical teams, provided offline and via virtual channels in other fields [10–12]. Prior research has provided a theoretical foundation relating to the impact factors of team outcomes. First, professional capital is generally divided into two dimensions, status capital and decisional capital, which have a significant influence on team performance [13] and doctors’ returns online, based on Social Exchange theory [14]. Second, team heterogeneity, relating to member compositions, affects performance directly and indirectly through various interactions [15, 16]. However, most studies have been conducted from the perspective of the individual doctor and ignore the other factors (social capital or team heterogeneity/diversity) in the team. Thus, further investigation is required into how MTs, as a new service delivery type, may attract more patients continuously.

Using data collected from a Chinese OHC, we empirically investigate how MTs can be deployed with maximum performance, based on the Upper Echelons Theory [16–18]. In light of the distinct characteristics of medical teams online, we explore the impact of the professional capital (status capital and decisional capital) of both the leader and medical team, as well as team heterogeneity and its moderating mechanisms. Specifically, this paper addresses the following key questions:

1. *How do the leader’s characteristics influence team composition?*
2. *How do the professional capital and team heterogeneity of medical teams affect the TSD?*

The rest of this paper is organized as follows. In Section 2, we critically review relevant literature and related theories. Then, hypotheses are presented in Section 3. In Section 4, we describe the research context, data collection methods, and variable measurement. Finally, we present and discuss our results, conclusions and implications for future research.

## Literature review

### Medical team strategy on OHCs and related theories

Collaboration among healthcare providers is proposed as a strategy for improving healthcare provision worldwide. Collaborative teams, operating offline, in healthcare scenarios, have become increasingly common in western countries [19, 20], and this trend is now moving towards the formation of online MTs [9]. Although there exists difficulties in working virtually, compared with traditional offline MTs [21–23], the emerging MTs on OHCs are seen to have the potential to improve the healthcare delivery due to the advantages mentioned above (specific and comprehensive). Second, online MTs disturb the traditional delivery method of ‘one-doctor-at-a-time’, and can be considered as a “group medical visit” [24]. Further, MTs offer the benefit of collective intelligence and the complementarity of time and knowledge or experience of other members, and provide team-based medical services without delay [25, 26]. As a new means of medical service provision, it is necessary to study the influencing factors of team-based service demands, which help promote its positive development and employment.

#### The upper echelons theory

This theory has explained the correlations between team outcomes and the background characteristics of teams, especially in superior management teams [18]. It provides a comprehensive framework for interpreting the effects of individuals and team attributes. The team founder (also called leader) takes advantage of their professional capital to build a team and attract patients who choose the team-based medical services on OHCs. Once a team is created, it has two characteristics: professional capital of the team and team heterogeneity. According to the Upper Echelons theory, the professional capital of a team leader and team heterogeneity reflect the strengths of the whole team, which could attract patient selection through reducing information asymmetry and ultimately contributing to team outcomes. To summarize, these characteristics of the team leader and team would affect the outcomes of most virtual teams [16, 27], however little is known about this phenomenon with relation to virtual teams operating in the healthcare industry.

### Professional capital and related theories

Professional Capital is considered a valuable and often rare type of ‘capital’ that is affiliated with social professionals, comprised of status and decisional dimensions as two motivations for consumption behavior [14, 28]. First, status capital manifests the individual and social advantage of professionals in society, which is always the basis for patients’ choices. In general, doctors’ titles, organizations (i.e., hospitals) and regions are applied to measure status capital in this paper. Second, decisional capital emphasizes the doctor-patient relationship, which mutually develops over a period of interactions [29], reflecting patient loyalty to the doctor.

#### The social exchange theory

The Social Exchange Theory has been widely used to understand exchanges on online communities [14]. Accordingly, interaction in MTs and between doctors and patients is a professional capital exchange, which indicates that teams with higher professional capital would result in providing more coherent team-based services. Obviously, it is mainly economic exchange between doctors and patients in the MTs mode.

Social capital has been extensively used at various levels, such as at an individual or collective level, involving communities, organizations and entire countries [30]. In this paper, capital is considered at both the individual and team level. At the leader level, the person plays a key role in the team and influence other members [31], so the professional capital of the leader will be considered in this paper. At the team level, Hongseok et al. demonstrated that a team’s social capital is the configuration of members’ social relationships and their structure [32], therefore the synthesized status and decisional capital of team members, and the decisional capital of the whole medical team is emphasized in this paper. According to social exchange theory, the professional capital of the leader and team would be bound to exert significant influences on the consumers’ choice.

### Team heterogeneity and its impact on team outcomes

Team heterogeneity commonly reflects the distribution of team member demographic characteristics, such as gender, age, functional experience, and tenure [16], which is also a vital feature of online MTs. Scholars have studied team heterogeneity from different perspectives. Some identified significant relationships between team heterogeneity (international experience, level of education, functional experience and tenure) and the outcomes of the team [15, 16], while others have investigated the moderating role of team heterogeneity [33, 34]. Thus, we explore the direct influence and moderating effects of team heterogeneity on team outcomes.

Based on the Upper Echelons Theory, team heterogeneity may influence team outcomes [33]. Prior research has not shown consistent conclusions regarding the relationship between team heterogeneity and outcomes. Some researchers have focused on the heterogeneity of demographic characteristics (e.g., education level, gender, age and experience of team members) and found positive relationships between team heterogeneity and team outcomes [35, 36]. However, different conclusions have been identified by other researchers [37], including one study which found that a team with high heterogeneity might increase contradiction and the cost of collaboration among members. With the emergence of MTs on OHCs, research is needed to examine the influence of team heterogeneity in the healthcare context.

### Research model and hypotheses

**Fig. 1:**
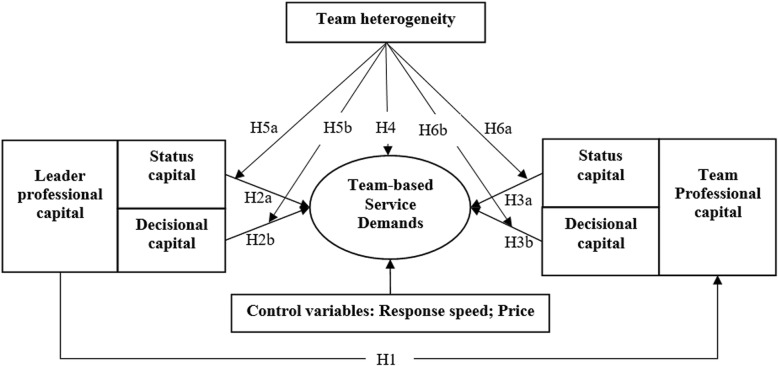
Conceptual Model

In this paper, we seek to examine the impact of the characteristics of both leaders and teams on the team-based service demands (TSD). Patients can seek and choose a MT on OHCs through either members’ homepages or the team’s homepage. Then they can obtain the service by scanning the Quick Response Code. Given secrecy and security requirements on OHCs, only price, response speed, and team composition is provided as available information to users. We extracted factors in this study from previous literature that focused on the impact of team heterogeneity on team performance in other fields and using related theories, especially the Social Exchange Theory, highlighting social or professional capital. This allowed for the construction of a conceptual model that comprises three main elements: (1) professional capital of team leader, (2) professional capital of the team, and (3) team heterogeneity. Details of the proposed model are shown in Fig. 1.

### Professional capital and TSD

We examine the influence of professional capital at both the team and individual level, based on prior research [38, 39], and the nature of the link between individual-level professional capital and team-level professional capital is described as follows. First, on OHCs, the professional capital of the team represents the collective value that a MT holds, regarding its interrelations and interconnectedness among members. Second, leadership is seen as essential for successful team operation [40, 41], and leaders of MTs have autonomy in selecting other members [42], typically motivated by outcome considerations rather than political or other concerns. Third, the professional capital of the leader plays an important role, and those leaders with high professional capital tend to attract members with high decisional capital (high loyalty and popularity). They also appeal to doctors with high status capital, as the leader usually assembles like-minded people. As a result, a team built by a leader with higher professional capital would experience stronger cooperation among members. Based on this analysis, the professional capital of the leader is highly and positively correlated to the professional capital of the team [43]. Thus, we hypothesize:

**H1: leaders with higher professional capital (status capital and decisional capital) are more likely to form teams with higher professional capital (status capital and decisional capital)**

Professional capital is considered in patient decision making. On the one hand, high status capital represents a greater amount of resources [44]. Patients treat a doctor with higher status (e.g., title or top-tier hospital) as a signal of higher service quality, believing that the doctor is better than others (i.e., more professional, knowledgeable, reliable and valuable) [11, 12]. On the other hand, a doctor’s popularity could be represented by decisional capital on OHCs [45]. It is commonly agreed that leadership is essential for team effectiveness and performance [46]. Leaders, whose key behavioral characteristic is dominance, have strong tendencies to “take the initiative in social settings, introduce people to one another, and be socially engaging by introducing and stimulating interaction” [47]. Popular doctors could help patients make good judgments on MTs and successful teams rely on their leader’s capacity [43]. Our study hypothesizes that the professional capital of a leader is significantly correlated with team outcomes:

**H2a(b): leaders with higher status capital (decisional capital) will have higher TSD**

**Extant research provides evidence concerning the positive effect of social/professional** capital embedded in a team on cooperation and team outcomes [48, 49]. Based on the Social Exchange Theory, professional capital, as a critical advantage, can enhance team functionality by influencing patients’ attitude and choices [50], so we confirm that a team with higher professional capital tends to obtain more patients. As the powerful synthesized professional capital of each team member works as a combined effect, patients tend to develop a psychological attachment to MTs through increasing perceived trust and lowered uncertainty from more than one doctor, which may directly contribute to patients’ intentions to repeatedly purchase services [51]. Specifically, status capital reflects the capability and level of members in offline MTs, while decisional capital indicates positive or negative relationships between doctors and patients online. In addition, patients may make choice based on peers [12], so the decisional capital of the whole team influences patient choice. Hence, users tend to trust MTs with high professional capital and purchase their services repeatedly. To summarize, the professional capital of teams contributes to team-based service demands on OHCs, leading to the following hypothesis:

**H3a(b): teams with higher levels of status capital (decisional capital) are more likely to receive more services in MTs**

### Team heterogeneity and TSD

In this study, team heterogeneity is reflected by team size (i.e., the number of team members) and dispersion of titles (i.e., Blau heterogeneity index [15] or the number of titles). Existing studies have proven that team heterogeneity could influence the outcomes or profitability of a team [16, 52], however, they do not have a consistent conclusion. This said, team size and dispersion (i.e., team heterogeneity) have consistently emerged as major antecedents of social loafing or productivity loss in technology-supported teams [53, 54]. For one reason, some scholars attribute the unsatisfactory outcomes to a dilution effect of the group member’s lessened ability in large-sized teams [55]. For another reason, users attach great importance to the professionalism (i.e., experience and knowledge) of members. In MTs with high title heterogeneity, members with an inferior title usually have more time and energy to provide services than ones with a superior title. Subsequently, patients are often worried that they may get the response from a less-experienced doctor, based on the theory of social loafing [53, 54], instead of the doctor that they want to choose, so patients are more willing to choose a homogeneous team.

The effect of team heterogeneity on outcomes also depends on the type of task and the development stage of the team [56]. The relationship between team heterogeneity and outcomes in related research manifests that: (1) heterogeneous teams are found to have advantages in unstructured, complex, short, creative and innovative work, and they are more productive in turbulent environments, and (2) by contrast, homogeneous teams are found to be more productive in stable environments, being good at long, procedural, regular and steady work [57]. Especially at the early development stage of a team, scholars have produced evidence that co-located teams outperform dispersed teams [58]. Team-based medical services often involve long and regular tasks [59], and MTs are at an early stage of development on OHCs in China. Therefore, heterogeneity in MTs may have a negative impact on TSD. This analysis leads us to the following hypothesis:

**H4: MTs with higher levels of team heterogeneity are more likely to possess lower team-based service demands in MTs**

The effects of leadership also depend on the specific environment, such as team heterogeneity and atmosphere. Team heterogeneity may enhance the role of leaders on outcomes by contributing to team openness and a diverse range of responses [60]. According to the uncertainty reduction theory [61], interaction with more than one doctor reassures patients, making them clearer about their symptoms or diagnosis received; this in-turn encourages repeat patient purchases. In addition, members of heterogeneous MTs have a complementary effect on each other, especially the leader, which is conducive for doctors when analyzing and solving problems, facilitating outcomes indirectly. In fact, patient choices on team-based medical services show the Matthew effect, that reflects cumulative advantage [62]. As a result, the mechanisms introduced above play critical roles in teams with leaders with higher professional capital than those with lower. Thus, we hypothesize the following:

**H5a(b): team heterogeneity positively moderates the relationship between the leader’s status capital (decisional capital) and TSD in MTs**

Following this logic, we argue that greater team heterogeneity encourages the sharing of different perspectives that helps embrace uncertainties [63, 64], thereby positively moderating the effect of a team’s professional capital on team based service demands. Doctors operating in a team deal with a patient’s questions simultaneously. Compared with a homogeneous team, a diverse MT will attach a higher value to the team with higher professional capital, which is more likely to reduce uncertainties and secure the trust of patients. High heterogeneity can solve problems more effectively and allocate time and energy of doctors more reasonably [34] by obtaining advice from colleagues with different expertise. Therefore, we suggest that the interaction between team heterogeneity and a team’s professional capital is conducive for the delivery of online healthcare and we hypothesize that:

**H6a(b): team heterogeneity positively moderates the relationship between a team’s status capital (decisional capital) and TSD in MTs**

## Methods

### Research context

The delivery of medical services by MTs on OHCs is an increasingly popular provision means in China. We study the research questions proposed in this paper by collecting data from haodf.com, considered one of the most professional and largest OHCs in China. The platform has developed sophisticated functionality, providing patients with the opportunity to purchase and obtain various medical services [65], including written consultation, phone consultation and video consultation. In 2017, Haodf.com introduced MTs to their platform for patient consultation. Since then, this new service has proved popular among both doctors and patients.

### Sample and data collection

**Fig. 2:**
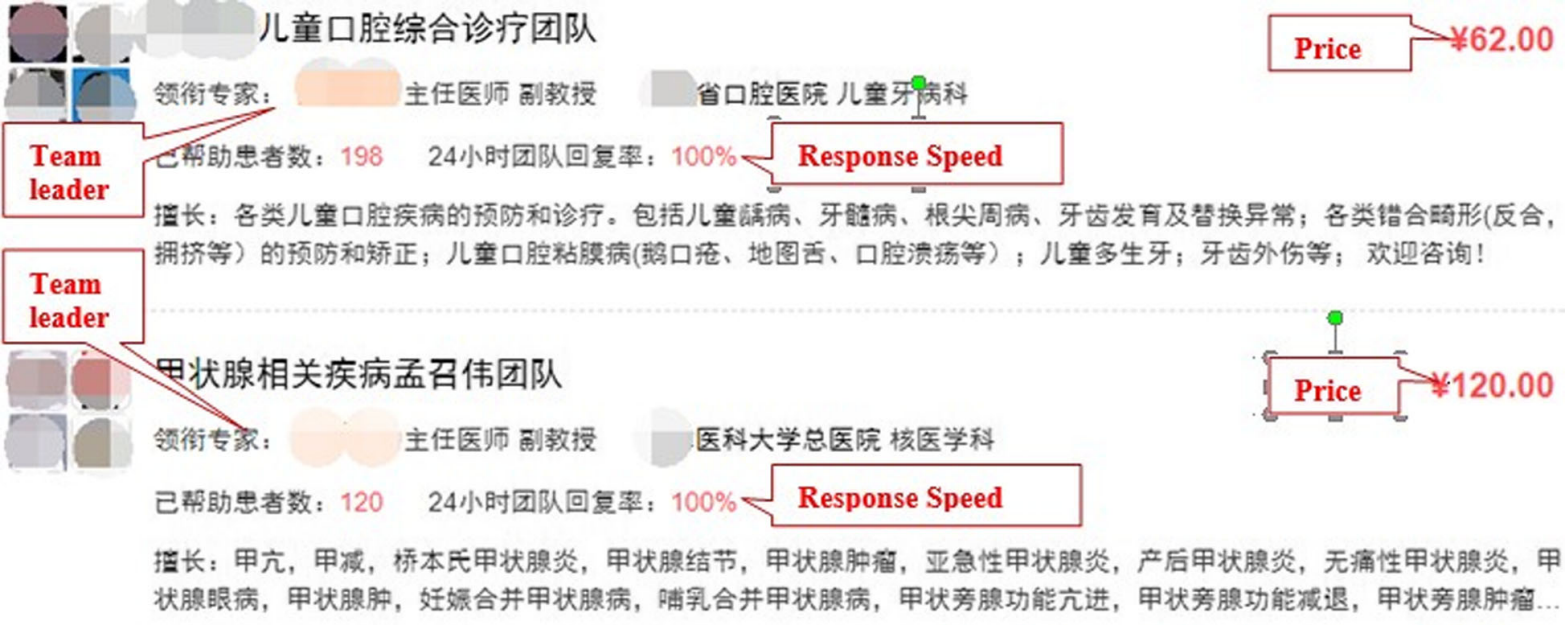
Service Selection Page of Team A and B

**Fig. 3:**
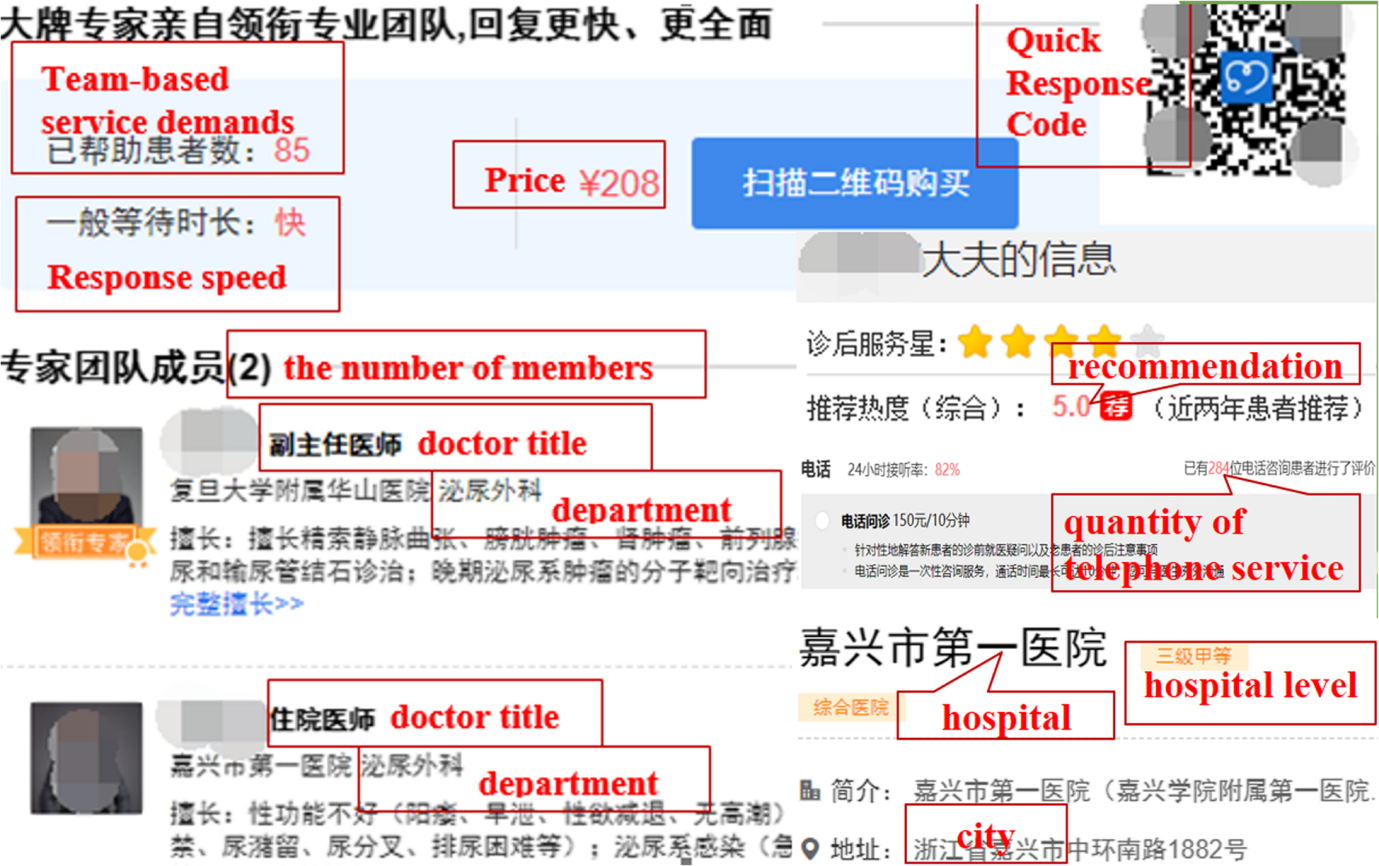
A MT’s Homepage

We crawled publicly available data and information relating to MTs on haodf.com to test our hypotheses. Data was collected for all teams on December 25, 2017, with the same process being repeated on January 25, 2018. As some important information, such as response speed, cannot be provided by MTs without patients, we removed these MTs from our dataset. Finally, 890 MTs, employing 3994 team members, were included in our model. For each team in our dataset, we collected data related to services offered, and other relevant information about the leader and team members (e.g., hospital and city information). Figure 2 shows an example of two teams’ online service selection pages, while Fig. 3 shows an example of a MT’s homepage. When a patient wishes to select a MT, they simply click the name of the team, as shown in Fig. 3, which provides a QR code allowing patients to scan the code to receive the team-based service via their mobile phone.

### Measures of variables and empirical models

#### Dependent variable

The dependent variable in this study was team-based service demands, which was measured by the quantity of patients who have purchased the team-based service. By collecting team information two times, we used the increment of *TSD* between December 25, 2017 and January 25, 2018 as the dependent variable.

#### Independent variables

Status Capital (*SC*) and Decisional Capital (*DC*) were included to measure professional capital and were used as two vital exchange resources. First, based on the research of Guo [14], the status capital of doctors was measured as follows: Clinic Title (*CTitle*), Academic Title (*ATitle*), Hospital Level (*HL*) and City Level (*CL*). We also assessed decisional capital by the doctor-patient interactions online: Recommendation (*Rec*) and Quantity of One-to-One Services (*Qoos*). Further, we conceptualized the variable of Team Heterogeneity (*TH*) by integrating two forms of diversity: Quantity of Team Members (*Qmem*) and *TH*, represented by Quantity of Titles (*Qtit*) or Blau Index (i.e., Herfindal-Hirschman Index) [15] of titles. Therein, the general formula of Blau Index was as follows,

1 $$ H=1-\sum \limits_{\mathrm{i}=1}{P_{\mathrm{i}}}^2 $$

where P_i_ was the proportion of each title in the number of team clinic titles. Finally, as the samples did not completely conform to standard normal distributions and each component had a different order of magnitude, we considered the standardization of all variables of *SC, DC,* and *TH*, to represent the primary data and address their large variances. Therefore, status capital was measured as the summation of four standardized variables: Clinic Title (*CTitle*), Academic Title (*ATitle*), Hospital Level (*HL*) and City Level (*CL*). Decisional capital was measured as the summation of two standardized variables: Recommendation (*Rec*) and Quantity of One-to-One Services (*Qoos*). Team Heterogeneity (*TH*) was measured as the summation of two standardized variables: Quantity of Team Members (*Qmem*) and title heterogeneity, represented by Quantity of Titles (*Qtit*) or Blau Index (i.e., Herfindal-Hirschman Index) [12] of titles. To summarize, the independent variables at the leader and team levels included Decisional Capital of Leader (*DCL*), Status Capital of Leader (*SCL*), Decisional Capital of Team (*DCT*), Status Capital of Team (*SCT*) and Team Heterogeneity (*TH*). A more detailed description of these is presented in Table 1, shown later in the article.

**Table 1:** Descriptive Statistics

Variables	Description/Explanation	Type	Mean	Std.	Min.	Max.
Dependent variables						
Team-based service demands (*TSD*)	The average number of team-based service demands the MT received during the two time periods.	Interval	1.958	4.6803	0	69
Independent variables						
Layer 1: Team Leader						
Decisional Capital (DCL)						
Recommendation (*Rec*)	Comprehensive recommended heat for the doctor, evaluated by patients.	Interval	4.144	0.382	3	5
Quantity of one-to-one service (*Qoos*)	The total number of one-to-one services that the doctor offers.	Interval	36.94	169.75	0	3166
Status Capital (SCL)						
Doctor’s Clinic Title (*CTitle*)	When clinic title of the lead doctor is Chief Doctor or Associate Chief Doctor, the value of *CTitle* is 1; otherwise, it is 0.	Dummy	–	–	0	4
Doctor’s Academic Title (*ATitle*)	When the academic title of the lead doctor is Professor or Associate Professor, the value of *ATitle* is 1; otherwise, it is 0.	Dummy	–	–	0	4
Hospital Level (*HL*)	Hospital level is evaluated and issued by government health departments. When hospital level is level 3, being the best, the value of *HL* is 1; otherwise, it is 0.	Dummy	–	–	0	3
City Level (*CL*)	When the team leader is in developed cities (Beijing/Shanghai/Guangdong), the value of *CL* is 1; otherwise, it is 0.	Dummy	–	–	1	4
Layer 2: MTs						
Decisional Capital (DCT)						
*Rec* of Team	Sum of recommendation of all members.	Interval	17.02	11.27	0	99
*Qoos* of Team	Sum of one-to-one service demands of all members.	Interval	49.82	182.05	0	3178
Patient Quantity (*PQ*)	The number of patients that the MT had at the beginning of this study.	Interval	13.99	25.273	0	640
Status Capital (SCT)						
Quantity with High Title Doctors (*DQHT*)	The number of doctors with clinic title of Chief Doctor or Associate Chief Doctor in the MT.	Interval	2.226	2.292	0	23
Quantity of Level3 Hospitals (*HQL*)	The number of doctors in Level3 hospital in the MT.	Interval	1.294	1.084	0	11
Quantity in Developed Cities (*DQDC*)	The number of doctors in developed cities in the MT.	Interval	2.06	2.876	0	24
Team Heterogeneity (TH)						
The quantity of Members (*Qmem*)	The number of member doctors in the MT.	Interval	4.488	2.900	2	24
Blau Index of Titles (*Blautit*)	Heterogeneity of member titles in the MT.	Interval	0.490	0.187	0	0.8
The quantity of Titles (*Qtit*)	The number of title types in the MT.	Interval	2.46	0.799	1	5
Control variables (Service Process)						
Response speed (*RS*)	Response speed of the MT for team-based written consultation.	Interval	0.433	0.436	0	1
Price (*P*)	Price offered by the MT for team-based written consultation.	Interval	73.62	83.56	0	800

#### Control variables

Where possible, we controlled for other factors, including response speed of services and price, which played a critical role in patient decision-making and ultimately affected team outcomes. First, online service providers could augment online service quality by enhancing their response speed, potentially attracting more patients [66]. Thus, for patients seeking online medical help from MTs, it was clear that response speed had a positive impact on TSD. Second, according to the transaction-cost theory, price affected quantity negatively, being regarded as the proxy of sacrifice made, so we controlled variables of response speed and price in our study to eliminate relevant effects.

All variables are described in detail in Table 1. We considered the logarithms of (*TSD* + 1) to deal with its large variances and the problem of the zero value. To measure the effect of professional capital and team heterogeneity respectively, we used hierarchical multiple regressions. We first controlled the effect of price and response speed on TSD in Model 1. Next, the professional capital of the team leader and the team were analyzed in Models 2 and 3. Finally, we further studied the impact of team heterogeneity and its interaction with professional capital on TSD. The coefficient of the interaction term between SCL/DCL/SCT/DCT and team heterogeneity would reveal whether team heterogeneity was statistically moderating the effect of professional capital on TSD. Our final empirical models were as follows, and *β*_*1*_*-β*_*11*_ represented the coefficient, *ε* represented the random error term .

2 $$ {\displaystyle \begin{array}{l}L\mathrm{n}\left( TSD+1\right)\times 10=\beta 1 RS+\beta 2P+\beta 3 DCL+\beta 4 SCL\\ {}+\beta 5 DCT+\beta 6 SCT+\beta 7 TH+\beta 8 DCL\times TH\\ {}+\beta 9 SCL\times TH+\beta 10 DCT\times TH+\beta 11 SCT\times TH+\varepsilon \end{array}} $$

## Results

Linear regression and hierarchical multiple regressions were used to estimate empirical results, while statistical significance was established at a *p*-value less than 0.05. All data was analyzed using STATA.

### Descriptive statistics and correlations

Descriptive statistics and Pearson’s correlation for the key variables used in the study are presented in Table 1 and Table 2 respectively. The minimum and maximum value of all variables is also shown in Table 1, while the range of TSD was from 0 to 65. The mean value for *TSD* was 1.958, which meant that each team had nearly two patients in each one-and-a-half month period, on average. In general, with relation to the low *TSD,* the skewness value of 7.405 indicated its positive skewness and the high standard deviation (i.e., 4.6803) represents its large variances, so it was necessary for us to clarify the variation in *TSD*, transform the variable of *TSD* as *Ln (TSD + 1)* and explore how professional capital and team heterogeneity influences it in MTs. The mean value (maximum value) for team size was 4.488 (21), with the mean value being low compared with the maximum value; therefore, MT heterogeneity and their impact on outcomes were to be explored. In addition, most correlations between the independent variables and control variables, shown in Table 2, were not high which helped yield stable results. The values of VIF were all below 10, so multicollinearity could be ignored [67].

**Table 2:** Bivariate Correlations (*n* = 890)

Variables	(1)	(2)	(3)	(4)	(5)	(6)	(7)	(8)	(9)	(10)	(11)	(12)	(13)	(14)	(15)	(16)
(1) *Rec*																
(2) *Qoos*	0.239^a^															
(3) *CTitle*	0.087^a^	0.102^a^														
(4) *ATitle*	0.200^a^	0.032	0.463^a^													
(5) *HL*	0.140^a^	−0.005	0.049	0.072^b^												
(6) *CL*	0.282^a^	0.142^a^	0.092^a^	0.023	−0.014											
(7) *Rec of Team*	0.155^a^	0.038	0.203^a^	0.126^a^	0.026	0.057										
(8) *Qoos of Team*	0.225^a^	0.894^a^	0.103^a^	0.000	−0.024	0.153^a^	0.157^a^									
(9) *PQ*	0.327^a^	0.570^a^	0.076^b^	0.021	0.013	0.128^a^	0.138^a^	0.585^a^								
(10) *DQHT*	0.058	0.033	0.335^a^	0.186^a^	0.021	0.038	0.714^a^	0.122^a^	0.049							
(11) *HQL*	0.094^a^	0.021	0.031	0.047	0.104^a^	0.071^b^	0.349^a^	0.048	0.073^b^	0.434^a^						
(12) *DQDC*	0.248^a^	0.113^a^	0.187^a^	0.162^a^	0.014	0.563^a^	0.505^a^	0.195^a^	0.154^a^	0.347^a^	0.195^a^					
(13) *Qmem*	0.100^a^	0.044	0.214^a^	0.120^a^	0.014	0.035	0.943^a^	0.131^a^	0.100^a^	0.756^a^	0.350^a^	0.450^a^				
(14) *Blautit*	0.046	0.071^b^	0.423^a^	0.218^a^	−0.021	0.098^a^	0.251^a^	0.084^b^	0.094^a^	0.130^a^	0.012	0.164^a^	0.262^a^			
(15) *Qtit*	0.109^a^	0.080^b^	0.428^a^	0.209^a^	−0.006	0.103^a^	0.511^a^	0.113^a^	0.128^a^	0.260^a^	0.078^b^	0.328^a^	0.518^a^	0.847^a^		
(16) *RS*	0.338^a^	0.101^a^	0.052	0.036	0.069^b^	0.105^a^	0.112^a^	0.149^a^	0.255^a^	0.024	0.050	0.095^a^	0.058	0.026	0.045	
(17) *P*	0.303^a^	0.120^a^	0.091^b^	0.097^a^	0.028	0.204^a^	0.134^a^	0.160^a^	0.013	0.109^a^	0.020	0.231^a^	0.091^a^	0.059	0.072^b^	−0.007

Notes: ^a^. Correlation is significant at the 0.01 level (2-tailed);^b^. Correlation is significant at the 0.05 level (2-tailed)

### Empirical results

The results relating to the relationship between the professional capital of the leader and professional capital of the team are shown in Table 3, which supports H1. All variables explained 31.1 and 16.2% variance in decisional capital and status capital, and the values of F test were all significant. For the decisional capital of the team, this was influenced significantly by that of the leader, and the leader’s clinical title. Thus, those leaders with high decisional capital were likely to be more loyal to provide services on OHCs and tended to choose similar doctors with higher perceived loyalty online. In addition, leaders with high clinical titles tended to attract similar types of team members. For the status capital of team, it had significant correlations with the leader’s status capital instead of decisional capital, that mainly referred to the leader’s behavior online. Specifically, the results indicated that leaders with high status capital often recruited team members with high status capital, which meant high titles (*CTitle, ATitle*), good hospitals (hospital level, *HL*) and developed cities (city level, *CL*), and this was consistent with the actual social relations. Finally, the decisional capital of the leader and the city level made most contributions to decisional capital and status capital of the team respectively, according to the standard coefficient.

**Table 3:** Results of Research Question 1

Variables	Decisional Capital		Status Capital	
	Coefficient	Standard coefficient	Coefficient	Standard coefficient
Intercept	−0.240		− 2.767^***^	
*CTitle*	0.507^***^	0.107	1.130^***^	0.120
*ATitle*	−0.137	−0.044	1.139^***^	0.185
*HL*	−0.143	−0.037	0.421^*^	0.055
*CL*	−0.016	−0.005	1.522^***^	0.253
*DCL*	0.536^***^	0.554	0.084	0.044
R^2^	0.311		0.162	
Adjusted R^2^	0.307		0.157	
F	79.754^***^		34.092^***^	

Notes: *N* = 890. * *p* < 0.1. ** *p* < 0.05. *** *p* < 0.01

All variables explained the 50.9% variance in *TSD*, and the values of R Square Change were all significant. To test the hypotheses of the proposed model, we considered five models. First, we only tested the effects of control variables (i.e. *RS* and *P*) in Model 1. Next, we added the professional capital of the team leader and MT in Model 2 and Model 3, where we evaluated H2 and H3. Furthermore, the impact of team heterogeneity on *TSD* (i.e., H4) was tested in Model 4. Finally, we built the interaction of professional capital and team heterogeneity to test H5 and H6. The regression equation estimation and results of these models are shown in Table 4. Through comparison of the adjusted R^2^ in each column, the five models all recorded satisfactory explanations, since R^2^ and ΔR^2^ were both significant.

**Table 4:** Results of Hierarchical Multiple Regression

Variables	Model 1		Model 2		Model 3		Model 4		Model 5	
	coefficient	Standard coefficient	Coefficient	Standard coefficient	Coefficient	Standard coefficient	Coefficient	Standard coefficient	Coefficient	Standard coefficient
Intercept	1.387^***^		1.229^***^		1.197***		0.972^***^		0.832^***^	
Control variables										
Response speed (*RS*)	4.134^***^	0.553	3.334^***^	0.446	3.198^***^	0.428	3.187^***^	0.427	3.215^***^	0.430
Price (*P*)	−0.003^**^	− 0.065	− 0.007^***^	− 0.178	− 0.006^***^	− 0.157	− 0.006^***^	−0.156	− 0.006^***^	− 0.154
Team leader										
*DCL*			0.727^***^	0.351	0.246^***^	0.119	0.190^**^	0.092	0.178^**^	0.086
*SCL*			0.296^***^	0.090	0.435^***^	0.132	0.480^***^	0.146	0.524^***^	0.159
MT										
*DCT*					0.551^***^	0.369	0.616^***^	0.412	0.605^***^	0.405
*SCT*					−0.053^***^	−0.096	−0.038^**^	−0.069	−0.045^*^	−0.082
*TH*							−0.181^***^	− 0.088	− 0.458^***^	−0.223
TH*DCL									0.091^**^	0.065
TH*SCL									0.133^**^	0.180
TH*DCT									−0.008	−0.012
TH*SCT									−0.005	−0.036
R^2^	0.311		0.436		0.496		0.501		0.509	
Adjusted R^2^	0.309		0.433		0.493		0.498		0.503	
F	200.208		170.868		144.928		126.386		82.817	
ΔR^2^	0.311		0.125		0.60		0.005		0.008	
ΔF	200.208		97.821		52.938		8.121		3.781	

Notes: *N* = 890. OLS present Ordinary Least Squares. * *p* < 0.1. ** *p* < 0.05. *** *p* < 0.01

In general, most paths were statistically significant, supporting hypotheses 1, 2, 4, and 5. Hypothesis 3 was partially supported, while H6 was not supported. Thus, the professional capital of a leader and team most positively and significantly influenced *TSD*; TH correlated negatively with *TSD*, which manifested that better teams were with lower variances in composition. Regarding the control variables in all models, price (*P*) was seen to have a negative impact on TSD, and response speed (*RS*) was shown to affect *TSD* positively. Furthermore, the control variable *RS* had the highest standard coefficient (0.427, *p* < 0.01) in Model 4, indicating that patients that use OHCs pay important attention to the response speed of MTs; thus, we could not ignore this variable.

Concerning the results of RQ2, we found that the professional capital of the leader and decisional capital of the whole team both had significant and positive effects on TSD. The path *DCT* → *TSD* (H3a) had the highest standard coefficient in the model (0.415, p < 0.01), except the control variable, indicating that *DCL* played a vital role. The results also suggested that *DCL*, *SCL,* and *DCT* had positive relationships with *TSD* (supporting H2a, H2b, and H3a). However, the status capital of the team did not function significantly, and H3b was not supported. Several factors contributed to it. First, considering that the primary form of team service was written consultation, and one-to-one services included both written and telephone consultation, there existed alternative and complementary effects between two service patterns, and TSD was influenced by individual service inevitably. Second, teams with high status capital tended to assign a high price to their services that could affect *TSD* significantly and negatively, concealing the influence of status capital of the team. Third, a team’s status capital partially mediated the effects of a leader’s professional capital on *TSD*.

### The effect of team heterogeneity

Team heterogeneity (*TH*) was seen to have complex roles, with the coefficient of heterogeneity on *TSD* being negative (β = − 0.458, *p* < 0.01, supporting H4), while a high positive interaction between heterogeneity and the leader’s professional capital was demonstrated. In detail, the coefficient of interaction between team heterogeneity and decisional capital of the leader was 0.091(*p* < 0.05), while that of the leader’s status capital was 0.133 (p < 0.05) (supporting H5). However, this association did not exist between heterogeneity and the team’s professional capital, so H6 was not supported. Accordingly, H2 (at the individual level) was supported, while H3 (at the team level) was only partially supported. In brief, it was imperative to research team outcomes at different levels, since it operated differently.

**Fig. 4:**
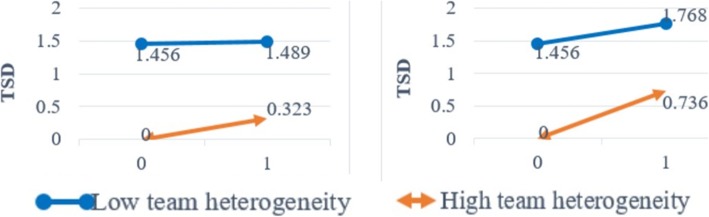
Interaction Effect between TH and Leader’s Professional Capital on TSD

The results obtained implied that the interaction effects between heterogeneity and leader’s professional capital at the individual level were significant for *TSD*, whereas no interaction occurred at the team level. Based on H5 and H6, we had investigated the potential moderating effect of team heterogeneity on the relationship between professional capital and *TSD*. Our results showed that a team’s heterogeneity enhanced the relationship between a leader’s social capital and e-consultation quantity, but it did not have any significant impact on the relationship between the team’s social capital and e-consultation quantity. A clearer representation of the interaction effects is shown in Fig. 4.

### Robustness test

We ran alternative model specifications by varying the proxies for heterogeneity. First, we used the number of titles in each MT as a proxy for title heterogeneity index. We combined the quantity of members and quantity of titles to represent the heterogeneity of the whole team by standardization and addition. The results were robust against these variations in the heterogeneity proxy, as shown in Table 5. Similarly, we used five models with their results being consistent with previous models. Thus, it was concluded that the results had no construct validity issues and were quite robust.

**Table 5:** Results of Robustness Test

Variables	Model 1		Model 2		Model 3		Model 4		Model 5	
	Coefficient	Standard coefficient	Coefficient	Standard coefficient	Coefficient	Standard coefficient	Coefficient	Standard coefficient	Coefficient	Standard coefficient
Intercept	1.387^***^		1.229^***^		1.197***		0.963^***^		0.785^***^	
Control variables										
Response speed (*RS*)	4.134^***^	0.553	3.334^***^	0.446	3.198^***^	0.428	3.184^***^	0.426	3.190^***^	0.427
Price (*P*)	−0.003^**^	− 0.065	− 0.007^***^	− 0.178	− 0.006^***^	− 0.157	− 0.006^***^	− 0.156	− 0.006^***^	− 0.155
Team leader										
*DCL*			0.727^***^	0.351	0.246^***^	0.119	0.181^**^	0.088	0.176^**^	0.085
*SCL*			0.296^***^	0.090	0.435^***^	0.132	0.466^***^	0.142	0.505^***^	0.154
MT										
*DCT*					0.551^***^	0.369	0.632^***^	0.423	0.620^***^	0.415
*SCT*					−0.053^***^	− 0.096	− 0.031	− 0.055	− 0.029^*^	− 0.053
*TH*							−0.203^***^	−0.108	− 0.445^***^	−0.238
TH*DCL									0.061^**^	0.055
TH*SCL									0.106^**^	0.168
TH*DCT									−0.008	−0.072
TH*SCT									−0.017	− 0.032
R^2^	0.311		0.436		0.496		0.502		0.511	
Adjusted R^2^	0.309		0.433		0.493		0.498		0.505	
F	200.208		170.868		144.928		127.239		83.296	
ΔR^2^	0.311		0.125		0.60		0.006		0.008	
ΔF	200.208		97.821		52.938		11.128		3.684	

Notes: *N* = 890. OLS present Ordinary Least Squares. * *p* < 0.1. ** *p* < 0.05. *** *p* < 0.01

## Discussion

### Result analysis

Given that prior research has examined factors that influence the outcomes of individual doctors on OHCs [12], and that limited studies have explored them in a team setting context, we propose a conceptual model in which we hypothesize and examine relationships between three characteristics (i.e., professional capital of leader and team, and team heterogeneity) and TSD in MTs settings based on the Social Exchange theory and Upper Echelons theory. In prior studies, the professional capital of doctors influences their outcomes positively [14]. Similarly, our results show that most leader (social capital and decisional capital) and team (decisional capital) characteristics have positive effects on TSD in MTs, while the status capital of a team has no significant effect. The results also show a negative relationship between team heterogeneity and TSD and the positive moderating effect of team heterogeneity on the relationship between the leader and TSD. We summarize the results of the estimation of hypotheses testing in Table 6. In short, our empirical results mostly support the hypotheses (i.e., the positive influence of most team characteristics) and possess decent explanatory power.

**Table 6:** Summary of Results

Hypotheses	Results
H1a. *CCL(+)➔CCT*	Supported
H1b. *SCL(+)➔CCT*	Supported
H1c. *CCL(+)➔SCT*	Supported
H1d. *SCL(+)➔SCT*	Supported
H2a. *CCL(+)➔TSD of MTs*	Supported
H2b*. SCL(+)➔TSD of MTs*	Supported
H3a. *CCT(+)➔TSD of MTs*	Supported
H3b. *SCT(+)➔TSD of MTs*	Not supported/significant
H4. *TH(−)➔TSD of MTs*	Supported
H5a*. CCL×TH(+)➔TSD of MTs*	Supported
H5b*. SCL×TH (+)➔TSD of MTs*	Supported
H6a*. CCT×TH (+)➔TSD of MTs*	Not supported/significant
H6b*. SCT×TH (+)➔TSD of MTs*	Not supported/significant

Note: The significant level of accepting/rejecting the hypotheses is at the 0.1 level

This research aids in the understanding of how a MT leader affects the composition of a team, and how these two aspects (leader and team) affect TSD. Scholars have tested how to maximize group effectiveness via optimal configurations of different conduits for such capital [32], and our results show that team based service demands could be increased by improving the composition of a MT. First, doctors with a higher service quality often have many duties in physical hospitals and have less time to concentrate on OHCs [65], so the complementary effect among doctors can improve efficiency and efficacy, and subsequently increase TSD. Second, if patients feel that a team’s heterogeneity is high while evaluating and comparing teams to make a choice, after basing their choice on the team leader, they become more willing to obtain services from the entire team because of increased perceived value. Thus, teams with a leader that possesses a higher professional capital could increase team heterogeneity to achieve a balance between service quality and response speed.

### Theoretical implications

Our study makes three contributions to theoretical understanding. First, we utilize the Upper Echelons Theory and Social Exchange Theory to frame our hypotheses and extend the literature on MTs. In particular, the extension of Upper Echelons theory, used for studying the influences of team characteristics on TSD of MTs, is a theoretical contribution for both those studying OHCs and for theory application. Meanwhile, existing studies regarding online healthcare communities are facing challenges of health information technology, consumer health informatics and incentives [65]; this study makes unique contributions to these aspects of health informatics in the context of MTs’ emergence and popularity.

Second, this study opens new avenues for research into online virtual teams, in the context of OHCs in China. On the one hand, it contributes to studies in related fields of team establishment and factors of team outcomes by studying teams in the healthcare field. It also makes numerous suggestions relating to team structure are provides insights into developing efficient MTs, which directly contributes to OHC research. On the other hand, we further extend the understanding of some conceptions. For instance, the concept of professional capital, widely used in organizational studies and OHC research, is expanded to team-based services on OHCs.

Finally, this research is distinctive from most other research, in that it analyzes the effect of team heterogeneity from two dimensions, instead of merely one dimension. Firstly, this study focuses on the direct impact of team heterogeneity, identifying different kinds of team heterogeneity for future research. Secondly, team heterogeneity, as a moderating variable, provides more comprehensive and powerful explanations for team outcomes from a novel perspective. Prior studies have postulated the influence of a team leader, while it has not been empirically examined in MTs, let alone the interaction effect of leader and team heterogeneity. The direct and indirect (i.e., moderating) roles of team heterogeneity in team outcomes helps us account for inconsistent conclusions in previous investigations.

### Practical implications

This paper has also made significant practical contributions. First, findings are deemed useful assets for healthcare providers, administrators and consumers in moving positively toward team-based services, which could help ensure the safety and quality of online health service provision. For healthcare providers, the results may allow MTs to understand what aspects should be given more attention for improving their services and competitiveness. For leaders, our findings offer evidence-based guidelines about the structural design of successful MTs on OHCs, while founders of teams should be cautious that high heterogeneity might not lead to envisaged outcomes. Furthermore, regarding managerial implications, the key to improving team performance in MTs is to consider the professional capital and team heterogeneity comprehensively; leaders with high professional capital could enhance team heterogeneity. Moreover, those teams with high heterogeneity should pay greater attention to reshaping team composition in the case of team loafing.

Finally, this research can help patients not only receive team-based services as an option of healthcare e-consultation, which removes the constraints of one-doctor-at-a-time services, but also informs them on how to choose MTs, improving communication efficacy and perceived service quality. The decisional capital of the whole team is more important for a user when choosing a MT, compared with the status capital of a highly reputed leader, according to the standard coefficients, and the decisional capital of team leader and the status capital of the whole team function least during patients’ decision-making process. In brief, the order of what patients focus on is: decisional capital of the whole team, status capital of team leader, decisional capital of team leader, and status capital of the whole team. Therefore, a patient may choose a team because the leader is highly reputed regardless of team members’ reputation, which means patients focus on the status capital of the leader more than the whole team.

### Limitations

Although this study makes significant contributions to theory and practice, it also has several limitations. First, the study uses cross-sectional data, instead of scientific panel data, which is taken from one OHC in China at the early stage of development when resources owned by MTs are in shortage, and there exists “New Entry Defects”, so continuous study is required, and future extensions could focus on comparative or comprehensive studies of one-on-one visits and MTs. In addition, we have not examined the cooperation mechanism for each team in our study. Further, there is medical information that cannot be integrated into the model. Patients with different symptoms and diseases typically ask for help from multiple departments, so the receivers of services would vary dependent on the specialties, symptoms, and diseases; in addition, this related information is difficult to obtain and measure. The final limitation relates to team heterogeneity. Although two aspects above could represent team heterogeneity well, according to related researches, there is a clear need for more work into the effect of different types of heterogeneity. The heterogeneity of background, department, hospital, and region could be taken into account to study the delivery of team-based services, just as Espinosa et al. [68] contended that the different types of team heterogeneity among virtual team members can have different effects on team process and outcome. Therefore, further longitudinal research is needed to explore these aspects to facilitate the improvement of service delivery.

## Conclusion

Demands of team-based service in OHCs have important practical significance in the healthcare domain, and they are strongly associated with the professional capital and team heterogeneity of MTs. In this paper, we comprehensively study the TSD from these two perspectives based on the Upper Echelons Theory and Social Exchange Theory, and results may assist the development of MTs in OHCs. First, concerning the professional capital, *DCL*, *SCL,* and *DCT* are key positive factors of TSD. Second, team heterogeneity has both significantly direct and indirect effects on TSD, since it negatively affects TSD and has a positive moderating effect on the relationship between the leader and TSD. According to the Contingency Theory and existing literatures, a contingent leader effectively applies their attributes dependent upon the internal/external conditions that include the team heterogeneity in this research. Thus, related suggestions are provided for the platform and team leaders to manage MTs with strong practicality.

## Data Availability

The datasets generated and/or analysed during the current study are not publicly available due to the privacy items of the Robots Protocol and since we collected the data from the haodf.com and would use them to write another paper, so data are not public, but are available from the corresponding author on reasonable request.

## Abbreviations

DC(L): Decisional Capital (of Leader)
DC(T): Decisional Capital (of Team)
MT(s): Medical Team(s)
OHC(s): Online Health Community(s)
OLS: Ordinary Least Squares
PC(L): Professional Capital (of Leader)
PC(T): Professional Capital (of Team)
SC(L): Status Capital (of Leader)
SC(T): Status Capital (of Team)
TH: Team Heterogeneity
TSD: Team-based Service Demands
